# Biological significance of monoallelic and biallelic *BIRC3* loss in del(11q) chronic lymphocytic leukemia progression

**DOI:** 10.1038/s41408-021-00520-5

**Published:** 2021-07-09

**Authors:** Miguel Quijada-Álamo, María Hernández-Sánchez, Ana-Eugenia Rodríguez-Vicente, Claudia Pérez-Carretero, Alberto Rodríguez-Sánchez, Marta Martín-Izquierdo, Verónica Alonso-Pérez, Ignacio García-Tuñón, José María Bastida, María Jesús Vidal-Manceñido, Josefina Galende, Carlos Aguilar, José Antonio Queizán, Isabel González-Gascón y Marín, José-Ángel Hernández-Rivas, Rocío Benito, José Luis Ordóñez, Jesús-María Hernández-Rivas

**Affiliations:** 1 University of Salamanca, IBSAL, IBMCC, CSIC, Cancer Research Center, Salamanca, Spain; 2 Department of Hematology, University Hospital of Salamanca, Salamanca, Spain; 3 Department of Medical Oncology, Dana-Farber Cancer Institute, Boston, MA 02115 USA; 4 Broad Institute of Harvard and MIT, Cambridge, MA 02142 USA; 5 Department of Hematology, Hospital Virgen Blanca, León, Spain; 6 Department of Hematology, Hospital del Bierzo, Ponferrada, Spain; 7 Department of Hematology, Hospital Santa Bárbara, Soria, Spain; 8 Department of Hematology, Hospital General de Segovia, Segovia, Spain; 9 Department of Hematology, Hospital Universitario Infanta Leonor. Universidad Complutense, Madrid, Spain; 10 Department of Medicine, University of Salamanca, Salamanca, Spain

**Keywords:** Chronic lymphocytic leukaemia, Cytogenetics

## Abstract

*BIRC3* is monoallelically deleted in up to 80% of chronic lymphocytic leukemia (CLL) cases harboring del(11q). In addition, truncating mutations in the remaining allele of this gene can lead to *BIRC3* biallelic inactivation, which has been shown to be a marker for reduced survival in CLL. Nevertheless, the biological mechanisms by which these lesions could contribute to del(11q) CLL pathogenesis and progression are partially unexplored. We implemented the CRISPR/Cas9-editing system to generate isogenic CLL cell lines harboring del(11q) and/or *BIRC3* mutations, modeling monoallelic and biallelic *BIRC3* loss. Our results reveal that monoallelic *BIRC3* deletion in del(11q) cells promotes non-canonical NF-κB signaling activation via RelB-p52 nuclear translocation, being these effects allelic dose-dependent and therefore further enhanced in del(11q) cells with biallelic *BIRC3* loss. Moreover, we demonstrate ex vivo in primary cells that del(11q) cases including *BIRC3* within their deleted region show evidence of non-canonical NF-κB activation which correlates with high BCL2 levels and enhanced sensitivity to venetoclax. Furthermore, our results show that *BIRC3* mutations in del(11q) cells promote clonal advantage in vitro and accelerate leukemic progression in an in vivo xenograft model. Altogether, this work highlights the biological bases underlying disease progression of del(11q) CLL patients harboring *BIRC3* deletion and mutation.

## Introduction

Chronic lymphocytic leukemia (CLL) patients harboring 11q22.3 deletion (del(11q)) are characterized by the presence of bulky lymphadenopathy, rapid disease progression and short time to first treatment (TTFT) and overall survival (OS) [1–4], even in early stage Binet A CLL cases [5]. The size of this deletion is heterogeneous, it can cover a region greater than 20 Mb in most of the patients, involving the loss of over a hundred genes [6]. The minimal deleted region almost always includes *ATM*, a putative CLL driver gene and one of the key components of the DNA damage response signaling [7, 8]. Another gene that has been hypothesized to also contribute to the pathobiology of del(11q) is *BIRC3*, which is located in the chromosomal band 11q22.2 and is entirely deleted in approximately 80% of del(11q) cases [9]. In addition, it has been shown that *BIRC3* disruption through truncating mutations occurs recurrently in CLL, ranging from frequencies of 3-5% in untreated cohorts to a two-fold higher incidence in relapsed/refractory CLL patients [10–13]. Interestingly, *BIRC3* mutations can appear in the remaining allele of approximately 10% of del(11q) patients with *BIRC3* monoallelic loss, resulting in a biallelic *BIRC3* inactivation [10, 12, 14]. Recent studies have shown that biallelic inactivation of *BIRC3* is an independent prognostic marker of inferior TTFT and OS in CLL [14, 15]. However, the clinical significance of *BIRC3* monoallelic mutations or deletion remains uncertain. Some studies have provided evidence of the clinical impact of *BIRC3* monoallelic mutations whereas others have not [10, 12, 14, 16–20]. Moreover, *BIRC3* mutations have also been found to be enriched in fludarabine relapsed/refractory CLL cases in some cohorts [10, 18], although the mechanistic insights by which *BIRC3* mutations could contribute to fludarabine resistance have not been elucidated.

Biologically, BIRC3 is known to have a role as a negative regulator of the non-canonical NF-κB signaling [21]. This pathway, alongside with the canonical NF-κB signaling, plays a key role on CLL pathogenesis, evolution and therapy response [22]. The non-canonical signaling is initiated by tumor necrosis factor (TNF) signals engaging B-cell activation factor receptor (BAFFR), CD40, lymphotoxin β-receptor (LTβR) or receptor activator for NF-κB (RANK) among others. In the absence of a stimulus, this pathway is kept inactive by the BIRC3-mediated ubiquitination and proteasomal degradation of NF-κB-inducing kinase (NIK). Upon receptor stimulation, BIRC3 is recruited to the active receptor complex and NIK is stabilized in the cytoplasm, promoting IKKα activation which in turn phosphorylates p100, leading to the proteasomal degradation of its C-terminus and the translocation of p52-RelB heterodimers into the nucleus to initiate NF-κB-dependent transcription [23]. In CLL, *BIRC3* mutations usually result in the loss of the E3 ubiquitin ligase domain essential for NIK targeting for proteasomal degradation, constitutively activating the non-canonical NF-κB signaling in a ligand-independent manner [10]. Nevertheless, the most frequent *BIRC3* alteration in CLL is monoallelic deletion of the entire gene through del(11q), being the functional consequences of this type of *BIRC3* monoallelic loss unexplored. In addition, it is unclear how biallelic *BIRC3* defects through del(11q) and *BIRC3* mutation in the remaining allele could contribute to a NF-κB-dependent acceleration of CLL progression.

The implementation of novel genomic editing technologies into the study of CLL has opened exciting possibilities to interrogate the functional effects of multiple driver genetic alterations as well as how some of these events cooperate to drive CLL progression and therapy response [24–27]. In this study, we used the CRISPR/Cas9 system to generate isogenic CLL-derived cell lines harboring del(11q) and/or *BIRC3* mutations in the remaining allele. We show that monoallelic *BIRC3* loss through del(11q) is enough to promote NIK-mediated non-canonical NF-κB signaling via p52-RelB nuclear translocation. Ex vivo experiments in primary del(11q) CLL cases revealed that del(11q) patients encompassing *BIRC3* within the deleted region had higher NIK levels as well as p52-RelB activity, which correlated with BCL2 overexpression. In addition, *BIRC3* loss-of-function mutations in del(11q) cells resulted in a higher activation of the non-canonical NF-κB signaling cascade, ultimately leading to increased clonal advantage in vitro and acceleration of leukemic progression in an in vivo xenograft model. Thus, our study provides novel biological insights about the role of *BIRC3* deletion and mutation in CLL evolution and progression.

## Methods

### CRISPR/Cas9-mediated engineering of CLL cell lines

HG3 and MEC1 Cas9-expressing cell lines (HG3-Cas9 and MEC1-Cas9) were previously generated and tested for Cas9 activity [27]. Single-guide RNAs (sgRNAs) targeting *BIRC3* (exons 2 or 7) were designed using the online CRISPR tool (http://crispr.mit.edu/), based on the predicted on-target efficiency and the lowest off-target effects. In addition, a sgRNA designed not to target the human genome was used as a negative control. SgRNAs targeting *BIRC3* were cloned into pLKO5.sgRNA.EFS.GFP (Addgene_#57822) plasmids and control sgRNAs were cloned in pLKO5.sgRNA.EFS.tRFP (Addgene_#57823). Sequences of the selected sgRNAs are detailed in Supplementary Table S1. The procedures and sgRNAs used for the generation of del(11q) and *TP53* mutations in HG3 cells were previously described [27, 28]. pLKO5 vectors carrying the desired sgRNAs were transduced into HG3-Cas9 and MEC1-Cas9 cells and single-cell flow-sorted clones were expanded and screened. At least three different clones harboring loss-of-function mutations were chosen for each CRISPR-generated cell line to perform functional studies.

### Primary CLL samples

Viably cryopreserved peripheral blood mononuclear cells (PBMCs) from 22 CLL patients were used in the ex vivo studies. PBMCs were isolated by Ficoll-Paque Plus density gradient media (GE Healthcare, Life Sciences) and a complete immunophenotypic analysis was performed in all samples by flow cytometry. Only samples with a CD19 + /CD5 + fraction greater than 85% were included in the study. Supplementary Table S2 summarizes the main biological characteristics of CLL patients. The research was conducted in accordance with the Declaration of Helsinki and prior approval by the Bioethics Committee from our institution. Written informed consent was obtained from all patients.

Next-generation sequencing (NGS) data from the primary CLLs used in the ex vivo experiments are detailed in Supplementary Table S3 and Supplementary Fig. S1. A custom NGS panel was applied and analyzed as previously reported [27, 29]. Full details about NGS procedure and analysis can be found in Supplementary Information.

### NF-κB family members activity ELISA

Canonical (p65/RelA, NF-κB1 p50, c-Rel) and non-canonical (NF-κB2 p52, RelB) NF-κB activity of nuclear extracts of HG3 and MEC1 clones and lysates from primary CLL samples was measured using the NF-κB Transcription Factor Assay Kit (Colorimetric) (Abcam, ab207216) following manufacturer’s instructions. Briefly, an oligonucleotide containing the NF-κB consensus site (5′-GGGACTTTCC-3′) has been immobilized onto a 96-well plate. Active NF-κB subunits present on the nuclear extracts specifically bind this oligonucleotide and p65, p50, c-Rel, p52, or RelB subunits are recognized by using specific primary antibodies accessible only when NF-κB is activated and bound to its target DNA.

### Ex vivo co-culture conditions

HS-5 stromal cells were seeded 24 h prior to the ex vivo experiments at a concentration of 7.5 × 10^4^ cells/well in a 6-well plate. On the following day, primary CLL cells were viably thawed and resuspended in RPMI 1640 medium (Life Technologies) supplemented with 10% FBS, 1% penicillin/streptomycin and 1.5 μg/mL CpG ODN (Sigma-Aldrich) plus 50 ng/mL IL-2 (Peprotech) and subsequently seeded onto the HS-5 cell layer at a co-culture ratio of 100:1 (7.5 × 10^6^ CLL cells /well) [30]. CLL cells were carefully detached and lysed 24 h after co-culture and proteins were subjected to NF-κB activity assays and/or immunoblot. CpG stimulation was chosen in order not to involve receptors directly implicated in non-canonical NF-κB activation such as CD40 or BAFFR.

### Xenograft experiments

Animal studies were conducted in accordance with the Spanish and European Union guidelines for animal experimentation (RD53/2013, Directive-2010/63/UE, respectively) and received prior approval from the Bioethics Committee of our institution.

For intravenous xenograft experiments, 20 four-to-five-week-old female NSG mice were used for injection of HG3 cells harboring del(11q) and/or *BIRC3* mutations (*n* = 5/each group). 3 × 10^6^ cells were resuspended in 100 μL of RPMI media and injected into the tail vein of the mice. 14 days after cell injection, mice were culled and spleens were subjected to FACS and immunohistochemistry analyses. For FACS analysis, spleens were lysed with erythrocyte lysis buffer, and the remaining cells were then washed twice in PBS. Samples were stained with fluorophore-conjugated antibodies against mouse-CD45 (PerCP-Cy5.5, BD Biosciences) and human-CD45 (hCD45) (CF Blue, Immunostep). Data were obtained on a FACSAria flow cytometer and analyzed with FlowJo software. Full details about subcutaneous xenografts in the Supplementary Information.

### Statistics

Statistical analyses were carried out using GraphPad Prism software v6 (GraphPad Software). Otherwise specified, data are summarized as the mean ± standard deviation (SD). Student’s *t* test, Mann–Whitney, ANOVA, or Kruskal–Wallis tests were used to determine statistical significance. *P*-values lower than 0.05 were considered statistically significant. At least three independent clones per condition were used in the functional studies.

### Supplementary methods

Supplementary Methods section includes detailed protocols of cell lines, culture conditions, drugs and reagents, NGS, FISH, subcellular fractionation and western blot, viability, apoptosis, and cell cycle analyses, in vitro clonal competition assays, subcutaneous in vivo xenografts and immunohistochemistry.

## Results

### CRISPR/Cas9-mediated generation of isogenic CLL cell lines harboring del(11q) and/or *BIRC3* mutations

**Fig. 1:**
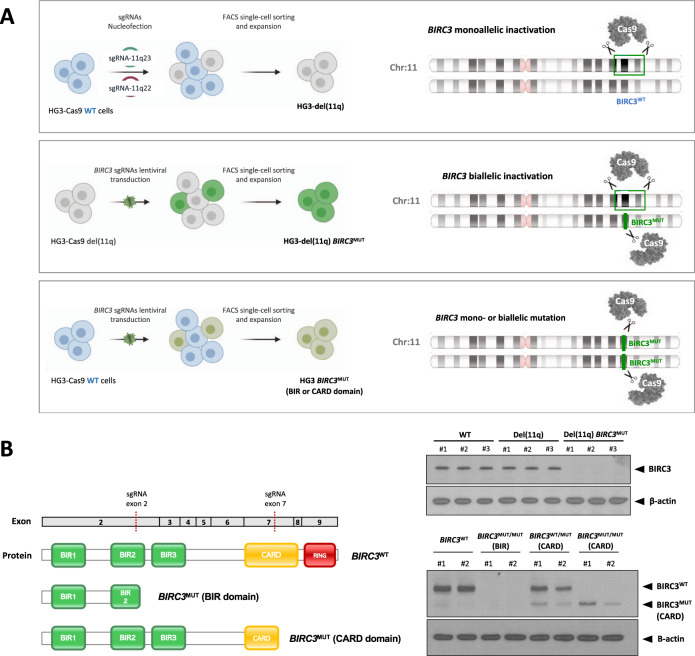
Generation of isogenic CRISPR/Cas9-edited CLL cell lines harboring del(11q) and/or *BIRC3* mutations. **A** Experimental design for the CRISPR/Cas9-mediated generation of *BIRC3*-related alterations in the Cas9-expressing HG3 CLL-derived cell line. Upper panel represents the design for monoallelic del(11q) (including monoallelic *BIRC3* loss) by the introduction of sgRNAs targeting 11q22.1 and 11q23.3. Middle panel displays the introduction of *BIRC3* mutations in HG3-del(11q) cells by the lentiviral transduction of a *BIRC3*-targeting sgRNA, generating HG3 cells with biallelic *BIRC3* loss through del(11q) and mutation in the remaining allele. Lower panel shows the steps required for the generation of *BIRC3* mutations (either in *BIRC3* exon 2 or exon 7) in HG3 cells without del(11q). The presence of del(11q) and/or *BIRC3* mutations was validated by FISH and Sanger sequencing, respectively. In total, at least three independent clones per condition were generated. **B** Left panel: BIRC3 WT and mutant protein diagram indicating the protein domains and the corresponding exons of the BIRC3 codifying sequence included in each *BIRC3*^MUT^-generated cell line. Right panel: BIRC3 western blot analysis of HG3-edited single-cell clones. β-actin was used as loading control.

In order to understand how monoallelic or biallelic *BIRC3* loss contributes to the pathobiology of del(11q) CLL, we used the CRISPR/Cas9-editing technology to model these alterations in an in vitro system. For this purpose, we selected HG3 and MEC1 CLL-derived cell lines. HG3 is diploid for chromosome 11 and has wild-type (WT) *BIRC3* gene. sgRNAs targeting chromosomal bands 11q22.1 and 11q23.3 were introduced in Cas9-expressing HG3 cells, generating an isogenic HG3 CLL cell line harboring a ∼17 Mb monoallelic del(11q) (HG3-del(11q)) encompassing *BIRC3* gene among others [27]. sgRNAs specifically targeting *BIRC3* (exon 2) were then introduced in HG3-del(11q) cells in order to induce *BIRC3* truncating mutations (*BIRC3*^MUT^) in the remaining WT allele, generating HG3-del(11q) *BIRC3*^MUT^ isogenic cell lines (Fig. 1A), mimicking the *BIRC3* biallelic loss through del(11q) and mutation observed in high-risk CLL patients. Furthermore, we also generated HG3 cell lines harboring only *BIRC3* mutation (HG3 *BIRC3*^MUT^) following the same strategy (Fig. 1A). *BIRC3* mutations were generated in a monoallelic or a biallelic fashion either in *BIRC3* exon 2 or exon 7, having as a consequence the truncation of the BIR or CARD BIRC3 protein domains, respectively (Fig. 1B, left panel), emulating the type of *BIRC3* mutations mainly detected in CLL [12, 16]. BIRC3 protein expression was evaluated by western blot in all the generated clones, showing that BIRC3 levels were absent in cells harboring truncating mutations in the exon 2 (BIR domain) or detecting a truncated form of BIRC3 in those clones harboring exon 7 mutations (CARD domain) (Fig. 1B; right panel).

In parallel, we used the MEC1 cell line as a model to study the *BIRC3* allelic dose effects in CLL. Parental MEC1 cells harbor a monoallelic *BIRC3* deletion (MEC1 *BIRC3*^DEL/WT^) as indicated by NGS copy number analysis (Supplementary Fig. S1). We also introduced sgRNAs targeting *BIRC3* (exon 2) following the previous approach, generating MEC1 cell lines harboring biallelic *BIRC3* loss through deletion and mutation (MEC1 *BIRC3*^DEL/MUT^) (Supplementary Fig. S2).

### *BIRC3* loss through del(11q) promotes p52-RelB nuclear translocation and activation of the non-canonical NF-κB signaling downstream targets

**Fig. 2:**
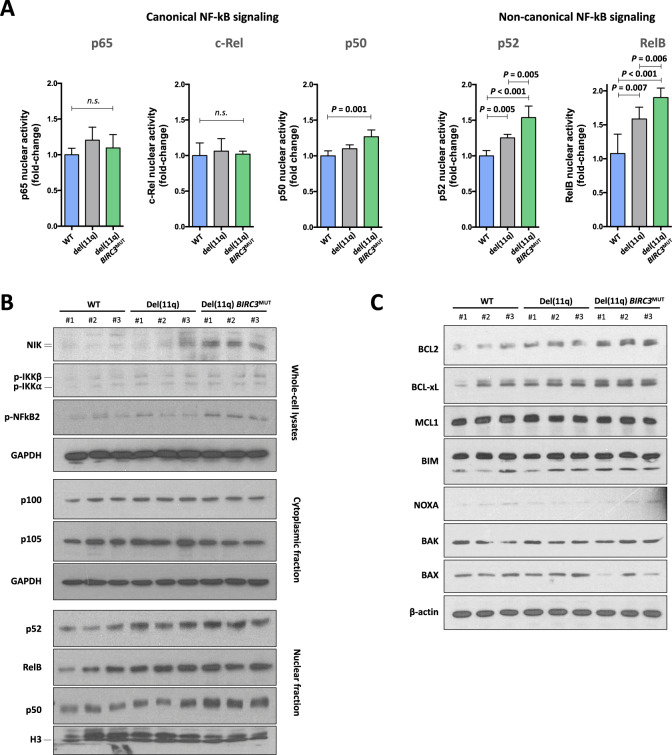
Evaluation of canonical and non-canonical NF-κB activity in del(11q)/*BIRC3*-deficient CRISPR/Cas9-engineered CLL cells. **A** ELISA measurement of relative NF-κB family transcription factor DNA-binding activity in nuclear extracts from HG3-edited clones. Left panel shows DNA-binding activity of NF-κB transcription factors involved in the canonical signaling (p65, c-Rel, and p50). Right panel displays the DNA-binding activity of non-canonical NF-κB transcription factors (p52 and RelB). Data are represented as the mean ± SD. **B** Whole-cell, cytoplasmic and nuclear lysates of HG3-del(11q) clones analyzed by immunoblotting for NIK, p-IKKα/β, p-NF-κB2, NF-κB2 (p100/p52), NF-κB1 (p105/p50), and RelB proteins. GAPDH was used as loading control for whole-cell and cytoplasmic lysates and H3 was used as loading control for nuclear extracts. Relative quantification for each protein (mean of three clones per condition) is depicted in Supplementary Fig. 3b. **C** Whole-cell lysates from HG3^WT^, HG3-del(11q), and HG3-del(11q) *BIRC3*^MUT^ analyzed by immunoblotting for BCL2 family members: BCL2, BCL-xL, MCL1, BIM, NOXA, BAK, and BAX. β-actin was used as loading control. Relative quantification for each protein (mean of three clones per condition) is detailed in Supplementary Fig. 4a.

Considering the role of *BIRC3* in the NF-κB signaling [21, 31], we assessed the impact of monoallelic and biallelic *BIRC3* loss through del(11q) and/or mutation in this pathway using our CRISPR/Cas9-engineered cell lines. We first analyzed the nuclear DNA-binding activity of the main NF-κB transcription factors implicated in both the canonical and non-canonical pathway. Regarding the canonical signaling, we did not observe significant changes in the activity of p65 and c-Rel. However, HG3-del(11q) *BIRC3*^MUT^ cells showed a significant increase of p50 nuclear activity in comparison to HG3^WT^ cells (Fig. 2A). Conversely, monoallelic *BIRC3* loss in HG3-del(11q) cells resulted in a marked increase of p52 and RelB activity, being this effect further enhanced in HG3-del(11q) *BIRC3*^MUT^ cells (Fig. 2A). These results were also confirmed in HG3 in all *BIRC3*^MUT^ clones (Supplementary Fig. S3a), confirming that either truncating mutations in the BIR or CARD domains have the same functional consequence on the non-canonical NF-κB signaling. Additional characterization of proteins involved in the non-canonical NF-κB signaling by western blot revealed that monoallelic, and to a greater extent, biallelic *BIRC3* loss resulted NIK cellular stabilization and increased levels of phosphorylated IKKα/β and NF-κB2, in line with a higher p52 and RelB accumulation in the nucleus (Fig. 2B; Supplementary Fig. S3b). Furthermore, we corroborated an increase of p50 nuclear levels in the nucleus of HG3-del(11q) *BIRC3*^MUT^ cells (Fig. 2B; Supplementary Fig. S3b).

To validate these results in an independent CLL cell line, NF-κB activity as well as NIK and p52 levels were analyzed in MEC1 cells. As expected, MEC1 *BIRC3*^DEL/MUT^ clones likewise presented higher p52, RelB and p50 activity rates, NIK stabilization and accumulation of p52 in the nucleus than MEC1 *BIRC3*^DEL/WT^ cells (Supplementary Fig. S3c, d).

Given that activation of the non-canonical signaling has been shown to upregulate some anti-apoptotic proteins such as BCL2 and BCL-xL [32, 33], we next assessed the protein levels of such these targets in our CRISPR/Cas9-edited cell lines in order to determine the impact of monoallelic or biallelic *BIRC3* loss in the regulation of these proteins expression. Interestingly, HG3-del(11q) *BIRC3*^MUT^ CLL cell lines showed higher levels of BCL2 and BCL-xL, alongside to reduced levels of pro-apoptotic protein BAX, whereas no changes were observed regarding MCL1 or pro-apoptotic family members such as BIM, BAK, and NOXA (Fig. 2C; Supplementary Fig. S4a). To test whether these increased levels of anti-apoptotic proteins were the result of BIRC3-mediated non-canonical signaling activation, cells were treated with a NIK small molecule inhibitor (NIK SMI1) [34], showing that NIK-dependent inhibition of p100/p52 processing translated into downregulation of BCL2 and BCL-xL protein expression in HG3-del(11q) *BIRC3*^MUT^ cells (Supplementary Fig. S4b).

### *BIRC3*-deleted primary del(11q) CLL cells show enhanced non-canonical NF-κB activity which correlates with high BCL2 levels

**Fig. 3:**
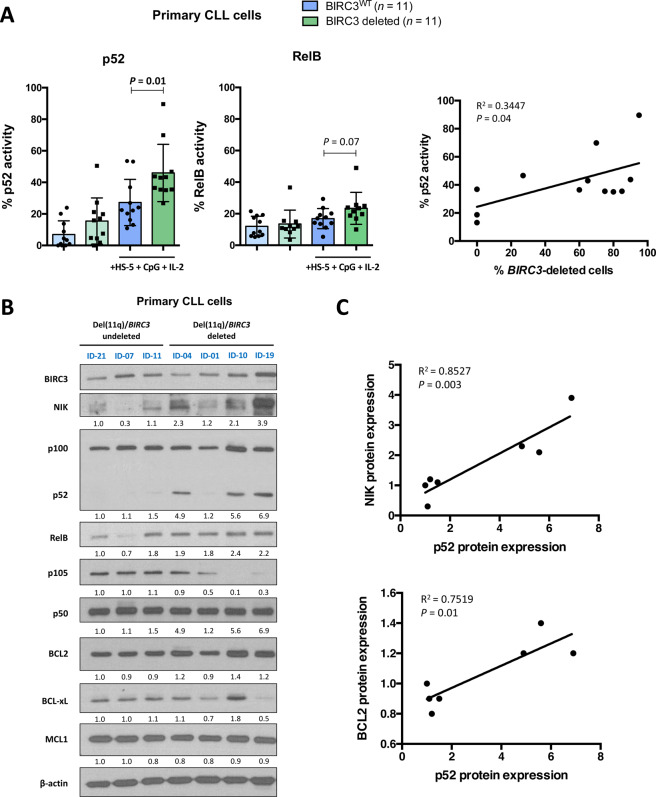
Effects of *BIRC3* loss in NF-κB activity and BCL2 levels of del(11q) primary CLL cells. **A** Left panel: ELISA measurement of relative NF-κB2 p52 and RelB DNA-binding activity in cell lysates from *BIRC3*^WT^ (including both non-del(11q) or del(11q) without *BIRC3* loss) (*n* = 11) and *BIRC3*-deleted (*n* = 11) primary CLL samples stimulated to proliferate with HS-5 cells, 1.5 μg/mL CpG and 50 ng/mL IL-2 or without stimulation. Proteins were extracted 24 h after co-culture. Data are represented as the mean ± SD. Right panel: correlation between the percentage of *BIRC3*-deleted cells in del(11q) patients (by integrating FISH and NGS data) and relative p52 activity taken from DNA-binding activity assays. Full details about cytogenetics and mutational status of the primary CLL cells used in the experiments are summarized in Supplementary Table S2. **B** Whole-cell lysates from stimulated CLL primary samples harboring del(11q) not involving *BIRC3* (del(11q)/*BIRC3*-undeleted) (ID-21, ID-07, ID-11) and del(11q) involving *BIRC3* (del(11q)/*BIRC3* deleted) (ID-04, ID-01, ID-10, ID-19) primary CLL samples were analyzed by immunoblotting for *BIRC3*, NIK, NF-κB2 (p100/p52), RelB, NF-κB1 (p105/p50), BCL2, BCL-xL and MCL1 proteins. β-actin was used as loading control. **C** Correlation between p52 protein levels and NIK (left panel) or BCL2 (right panel) protein levels from the patients analyzed by immunoblot.

In order to validate whether the results obtained in our CRISPR/Cas9-generated models could resemble the actual biology of *BIRC3*-deleted del(11q) CLL patients, we tested the DNA-binding activity of non-canonical NF-κB transcription factors in a cohort of 22 CLL cases (*n* = 11 *BIRC3*^WT^; *n* = 11 *BIRC3*-deleted through del(11q) or mutation) (Supplementary Table S2) in the absence or presence of stromal + CpG + IL-2 stimulation. Remarkably, stimulated *BIRC3*-deleted CLL cells showed higher p52 activity than *BIRC3*^WT^ cases (Fig. 3A; left panel), in line with the results observed in HG3-del(11q) cells. To a lesser extent, *BIRC3*-deleted cases also showed a trend of higher RelB activity than *BIRC3*^WT^ cells (Fig. 3A, left panel). In addition, focusing on the subgroup of patients harboring del(11q), we could observe a significant correlation between the percentage of *BIRC3*-deleted cells and p52 activity (Fig. 3A, right panel), further evidencing the NF-κB-related effect of *BIRC3* monoallelic loss in del(11q) cases.

Next, we performed western blot analyses in a homogenous cohort of del(11q) samples including or not including *BIRC3* within the deleted region (*n* = 4, del(11q)/*BIRC3* deleted; *n* = 3, del(11q)/*BIRC3* undeleted). Interestingly, del(11q)/*BIRC3* deleted cases presented high levels of stabilized NIK, resulting in a marked NF-κB2 p52 processing, which was virtually absent in del(11q)/*BIRC3* undeleted cases (Fig. 3B). Indeed, there was a clear correlation between NIK and p52 levels in these patients (Fig. 3C, upper panel). Of note, del(11q)/*BIRC3* deleted cases also showed increased levels of RelB and a reduction of NF-κB1 p105 precursor levels, although we did not observe differences in p50 protein expression between these groups (Fig. 3B). In addition, among anti-apoptotic BCL2 family members, del(11q)/*BIRC3* deleted cases showed higher BCL2 protein expression than del(11q)/*BIRC3* undeleted cases (Fig. 3B), which correlated to the amount of p52 levels in these patients (Fig. 3C, lower panel).

### Biallelic *BIRC3* loss confers sensitivity to BCL2 and BCL-xL inhibition in vitro

**Fig. 4:**
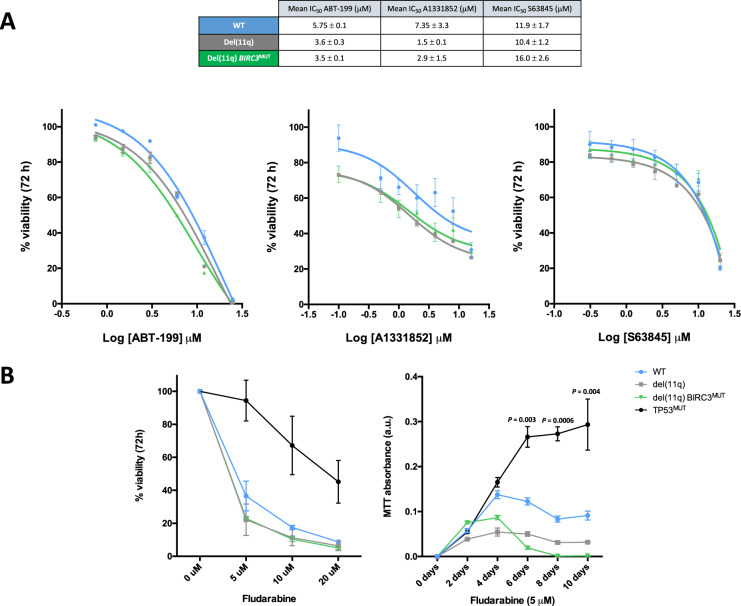
Cell viability studies of del(11q)/*BIRC3*-mutated cell lines in response to BCL2, BCL-xL, MCL1 inhibition, or fludarabine treatment. **A** Dose-response curves of HG3^WT^, HG3-del(11q) and HG3-del(11q) *BIRC3*^MUT^ clones treated with venetoclax (ABT-199; BCL2 inhibitor, left panel), A1331852 (BCL-xL inhibitor; middle panel) and S63845 (MCL1 inhibitor, right panel). Cell viability was assessed by MTT assay after 72 h and surviving fraction is expressed relative to DMSO control. Data are summarized as mean ± SEM. **B** Left panel: HG3-edited clones were treated with escalating doses of fludarabine and cell viability was assessed by MTT assay after 72 h. Surviving fraction is expressed relative to DMSO control. Data are summarized as the mean ± SD. Right panel: HG3-edited clones were treated with fludarabine at a concentration of 5 μM. Cell viability was assessed by MTT every 2 days up to 10 days. Proliferation rates are presented as MTT absorbance units, and data are shown as mean ± SD. *P*-values indicate differences between HG3^WT^ and HG3 *TP53*^MUT^ clones.

Considering the effects of *BIRC3* loss in the upregulation of some anti-apoptotic family members, we next evaluated the response of the isogenic CRISPR/Cas9 HG3 clones to selective BCL2, BCL-xL or MCL1 inhibition. BCL2 inhibition with venetoclax (ABT-199) highlighted a higher sensitivity of HG3-del(11q) *BIRC3*^MUT^ cells than HG3^WT^ cells (Fig. 4A), in line with the observed non-canonical NF-κB-dependent BCL2 upregulation of these cell lines. In addition, HG3-del(11q) and HG3-del(11q) *BIRC3*^MUT^ cells were also more sensitive to BCL-xL inhibition by A1331852 than HG3^WT^ cells (Fig. 4A). Contrarily, monoallelic or biallelic *BIRC3* loss in HG3 cells did not seem to influence the response to MCL1 inhibition by S63845 (Fig. 4A), consistently with our observations regarding MCL1 protein levels. Furthermore, we also tested the response of these cell lines to the BTK inhibitor ibrutinib, showing that HG3-del(11q) *BIRC*3^MUT^ cells were slightly less sensitive in comparison to HG3^WT^ cells (Supplementary Fig. S5a).

Moreover, since *BIRC3* disruption in CLL patients has been associated with fludarabine refractoriness even in *TP53* wild-type CLLs [10], we tested HG3-del(11q) clones (with or without *BIRC3* disruption) for evidence of resistance to fludarabine treatment. HG3 *TP53*^MUT^ clones, also generated by CRISPR/Cas9, were used as positive controls for fludarabine resistance. Interestingly, after 72 h of fludarabine treatment, only *TP53*^MUT^ clones presented marked resistance (by MTT assay) whereas HG3-del(11q) *BIRC3*^MUT^ clones showed the same sensitivity as HG3^WT^ cells (Fig. 4B, left panel). Longer drug exposures were also tested, and we found no significant resistance of *BIRC3* disrupted clones (Fig. 4B, right panel). Further support for fludarabine treatment-induced apoptosis in *BIRC3*-deficient clones was observed through the appearance of a sub-G_0_ peak in cell cycle profiles and annexin studies (Supplementary Fig. S5b, c).

### Biallelic *BIRC3* loss in del(11q) CLL cells favors clonal advantage in vitro

**Fig. 5:**
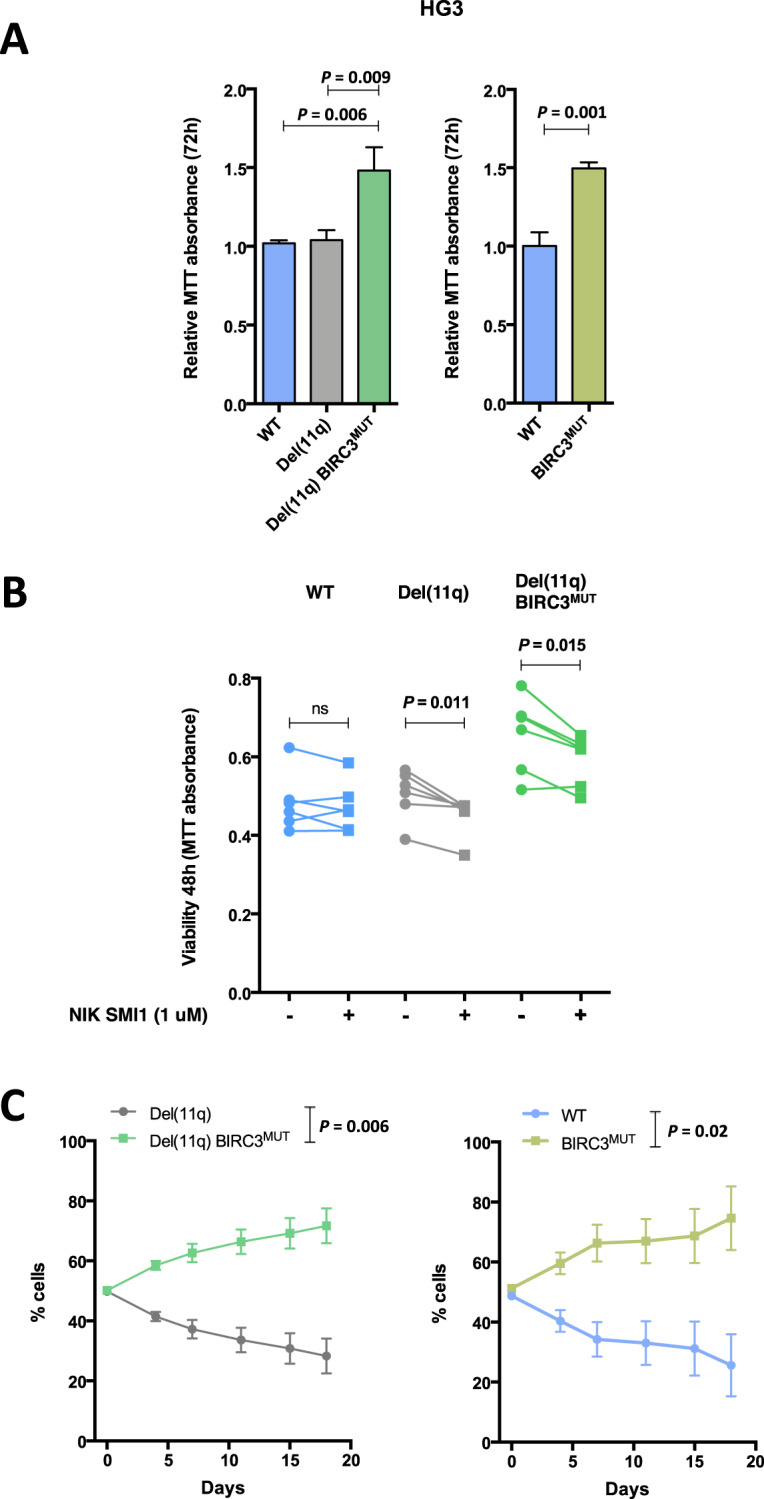
Effects of del(11q) and/or *BIRC3* mutations in CLL cell lines proliferation and clonal evolution. **A** Analysis of del(11q) and/or *BIRC3* mutations on proliferation of HG3 cells after 72 h. MTT absorbance values are normalized with the HG3^WT^ clones. Data are summarized as the mean ± SD. **B** Viability analysis of HG3^WT^, HG3-del(11q), and HG3-del(11q) *BIRC3*^MUT^ cells treated with DMSO or 1 μM NIK SMI1. Cell viability was assessed by MTT assay after 48 h and data is represented as the absolute absorbance value for each independent clone. **C** HG3-del(11q) RFP-tagged and HG3-del(11q) *BIRC3*^MUT^ GFP-tagged cells (left panel), HG3^WT^ RFP-tagged and HG3 *BIRC3*^MUT^ GFP-tagged cells (right panel), were mixed at a ratio 1:1 and left in culture for the indicated days. Clonal evolution was assessed at the indicated time points by flow cytometry. Bars represent mean ± SD.

We next hypothesized that the effects of *BIRC3* loss in the NF-κB signaling and apoptosis may have an impact on CLL evolution and progression. For this purpose, proliferation assays were performed to characterize the consequences of the CRISPR/Cas9-generated alterations in CLL expansion. We noted that HG3-del(11q) *BIRC3*^MUT^ cells showed enhanced viability and growth than HG3-del(11q) and HG3^WT^ cells (Fig. 5A, left panel; Supplementary Fig. S6a). In addition, cell cycle analyses of these clones revealed that HG3-del(11q) *BIRC3*^MUT^ cells had a higher proportion of cells transitioning through S-phase (Supplementary Fig. S6b). To test whether this effect on proliferation could be attributed to *BIRC3* loss, MTT and growth assays were carried out in HG3 *BIRC3*^MUT^ cells without del(11q), and these indeed confirmed the higher proliferation rates of *BIRC3*-deficient cells (Fig. 5A, right panel; Supplementary Fig. S6a). Moreover, HG3^WT^ cells treated with the BIRC2/BIRC3 inhibitor birinapant also displayed increased growth (Supplementary Fig. S6c) as well as MEC1 *BIRC3*^DEL/MUT^ cells in comparison to MEC1 *BIRC3*^DEL/WT^ (Supplementary Fig. S6d). We also determined that this enhanced proliferation rate was driven by enhanced BIRC3-mediated non-canonical NF-κB signaling activation, since NIK inhibition by SMI1 was able to reduce viability of HG3-del(11q) and HG3-del(11q) *BIRC3*^MUT^ cells (Fig. 5B).

In order to evaluate how *BIRC3* deletion and/or mutation could contribute to CLL clonal dynamics, we next carried out in vitro clonal competition experiments by mixing RFP- or GFP-tagged CRISPR/Cas9-edited cells at a ratio 1:1 and tracked clonal evolution overtime by flow cytometry. In the first experiment, clonal competition was assessed to investigate how *BIRC3* mutation could confer a clonal advantage of del(11q) cells. Notably, HG3-del(11q) *BIRC3*^MUT^ cells progressively outgrew HG3-del(11q) cells overtime (Fig. 5C). In a second experiment, we evaluated the clonal competition between HG3^WT^ and HG3 *BIRC3*^MUT^ cells, showing that HG3 *BIRC3*^MUT^ cells were able to outcompete HG3^WT^ cells (Fig. 5C).

### Biallelic *BIRC3* loss in del(11q) CLL cells accelerates leukemic progression in in vivo xenografts

**Fig. 6:**
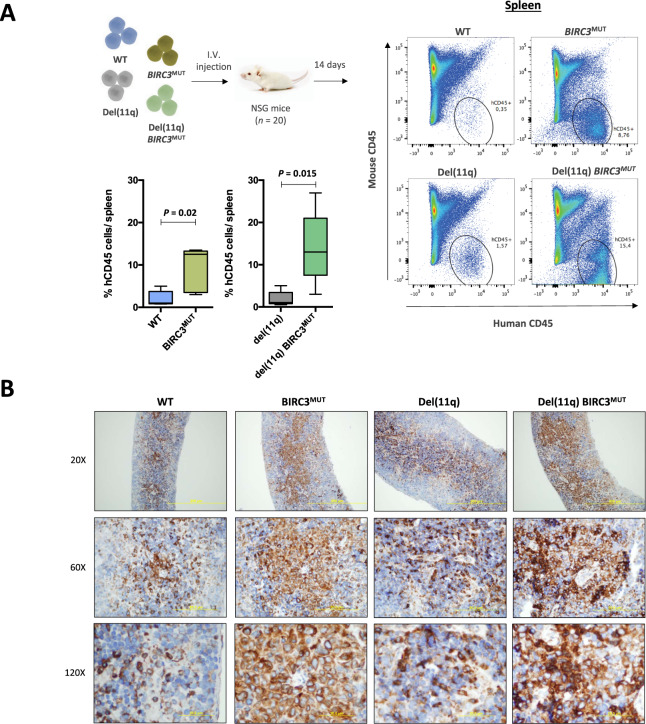
In vivo analysis of leukemic progression of del(11q)/*BIRC3*-mutated CRISPR/Cas9-edited clones. **A** Spleen infiltration of xenotransplanted HG3^WT^, HG3 *BIRC3*^MUT^, HG3-del(11q), and HG3-del(11q) *BIRC3*^MUT^ cell lines (*n* = 5/per group) into NSG mice. Mice spleens were analyzed by FACS 14 days after cell injection and hCD45+ cells were monitored to evaluate the leukemic infiltration in each condition. **B** Immunohistochemical analysis of NF-κB2 (p52) expression in spleens of HG3^WT^, HG3 *BIRC3*^MUT^, HG3-del(11q), and HG3-del(11q) *BIRC3*^MUT^ xenografted mice.

To confirm the effects of *BIRC3* loss in a physiological context in vivo, we individually injected the monoallelic and biallelic *BIRC3*-deleted CRISPR/Cas9-edited cell lines intravenously into NSG mice, observing that mice xenografted with HG3 *BIRC3*^MUT^ and HG3-del(11q) *BIRC3*^MUT^ cells showed an increase of human CD45+ cells in spleen 14 days after injection, compared to HG3^WT^ and HG3-del(11q) cells, respectively, by flow cytometry (Fig. 6A). By immunohistochemistry, spleens collected from HG3 *BIRC3*^MUT^ and HG3-del(11q) *BIRC3*^MUT^ intravenous xenografted cells propagating in vivo also showed evidence of NF-κB2 activation (Fig. 6B).

In addition, to validate this effect in the proliferation, HG3^WT^ and HG3 *BIRC3*^MUT^ cells were injected subcutaneously in the flank of NSG mice and tumor growth was monitored for 17 days. HG3 *BIRC3*^MUT^ cells generated larger tumors than HG3^WT^ cells (Supplementary Fig. S7a). Besides, tumors collected from HG3 *BIRC3*^MUT^ engrafted mice showed higher levels of p52 expression than those from HG3^WT^ mice (Supplementary Fig. S7b).

## Discussion

Del(11q) is one of the most frequent cytogenetic abnormalities occurring in CLL patients [4, 35, 36], yet, the functional consequences of the haploinsufficiency of the vast majority of genes comprised within this region remains largely unknown. Here, we undertook a CRISPR/Cas9-based genome editing approach to characterize novel biological implications of monoallelic and biallelic *BIRC3* loss in del(11q) CLL. In this way, our work presents in vitro, ex vivo, and in vivo evidence of how *BIRC3*-deletion and/or mutation in the remaining allele of del(11q) cells contributes to NF-κB signaling activation, CLL progression, and therapy response.

Our data indicate that monoallelic *BIRC3* deletion contributes to the pathobiology of del(11q) by a NIK-dependent triggering of the non-canonical NF-κB signaling, resulting in enhanced p52-RelB nuclear translocation and activation (Supplementary Fig. S8). This effect appears to be allelic dose-dependent since biallelic *BIRC3* loss resulted in higher activation rates (Fig. 2). Our results obtained in isogenic CLL-derived cell lines provide a more comprehensive landscape of the role of each CLL *BIRC3*-related alterations in the non-canonical NF-κB pathway, complementing previous findings hinted in a panel of lymphoid-related cell lines [18]. In addition, we were also able to address the biological differences between del(11q) CLL patients including or not *BIRC3* within their deleted region, showing that, in response to stromal stimulation and TLR ligation by CpG, del(11q)/*BIRC3*-deleted cases present marked levels of stabilized NIK and p52 activity. Indeed, further evidence of hyperactive non-canonical signaling was found in B-lymphocytes from mice lacking *cIap1*/*cIap2* (*Birc2*/*Birc3*) [37] and in B-cells treated with BIRC2/BIRC3 inhibitors [21], as well as in our isogenic del(11q) CLL cell lines (Fig. 2). Furthermore, a previous report observed that CLL cells with low *BIRC3* mRNA expression presented activation of the canonical NF-κB signaling in the presence of BAFF or CD40L stimulation [31]. Interestingly, we did find partial evidence of canonical NF-κB activation by increased nuclear p50 activity in HG3-del(11q) *BIRC3*^MUT^ cells, which has also been shown to contribute in the pathogenesis of Eμ-TCL1 model of CLL [38]. This enhanced p50 activity could be in line with the high phosphorylation levels of IKKβ, a member of the IκB-kinase (IKK) complex, implicated in canonical NF-κB activation [39]. Altogether, these results suggest that, in the presence of the CLL microenvironment, *BIRC3* loss displays a dual role on both canonical and non-canonical NF-κB signaling activation.

The recent introduction of the selective BCL2 inhibitor venetoclax into the CLL treatment scheme has led to effective remissions for relapsed/refractory CLL patients, especially when combined with anti-CD20 antibodies [40, 41]. Nevertheless, little is still known regarding which genetic alterations may predict for better venetoclax responses in CLL. We show that enhanced non-canonical NF-κB activity in *BIRC3*-deleted cells results in BCL2 overexpression, making isogenic del(11q) *BIRC3*^MUT^ cells more sensitive to venetoclax treatment. These observations are limited due to the use of CLL cell lines which do not display the same venetoclax sensitivity as primary CLL cells [42]. However, we also observe a correlation between BCL2 levels and the percentage of *BIRC3*-deleted cells in del(11q) cases, as well as between p52 and BCL2 levels, suggesting that *BIRC3*-deleted cases may potentially benefit from venetoclax-based regimes. In fact, recent data from the CLL14 trial suggest that del(11q) or *BIRC3*^MUT^ patients significantly favor from venetoclax plus obinutuzumab in comparison to the chlorambucil plus obinutuzumab treatment arm [43]. In addition, we show that NIK pharmacological inhibition can counteract BIRC3-mediated non-canonical NF-κB signaling and anti-apoptotic protein overexpression, making it an attractive candidate for combinatorial therapy with venetoclax [44]. Contrarily, del(11q) *BIRC3*^MUT^ cells did not selectively benefit from ibrutinib treatment, in agreement with previous reports indicating that BTK inhibition does not suppress non-canonical NF-κB signaling activity [18, 45]. Furthermore, we also assessed the treatment implications of *BIRC3* deletion and/or mutation in response to fludarabine, given that these alterations have been associated with fludarabine relapse in some, but not all, cohorts [10, 12, 18]. Nonetheless, neither isogenic HG3-del(11q) *BIRC3*^MUT^ nor *BIRC3*^MUT^ cells show evidence of fludarabine resistance, whereas isogenic HG3 *TP53*^MUT^ cells present marked resistance in the same conditions (Fig. 4B). These results indicate that *BIRC3* alterations may not be enough to generate fludarabine resistance per se, as opposed to *TP53* alterations. Further investigation is required to decipher whether extrinsic factors such as the CLL microenvironment as well as the concurrence with other genetic alterations would play a critical role in fludarabine resistance of *BIRC3* mutated CLL cells.

Although there is still controversy regarding clinical impact of *BIRC3* mutations, recent reports have highlighted the negative predictive impact on TTFT and OS of biallelic *BIRC3* loss through del(11q) and mutation in the remaining allele [14, 15]. Our work biologically demonstrates that biallelic *BIRC3* loss promotes CLL proliferation, clonal evolution and progression in vitro and in vivo. These results are further supported by the notion that mice lacking *cIap1*/*cIap2* show an uncontrolled accumulation of B-cells in vivo [37]. Interestingly, we did not observe the enhanced proliferation of isogenic cell lines harboring monoallelic *BIRC3* loss, suggesting that *BIRC3* mutations may only have a clinical impact in patients with a previous del(11q) background. These data therefore reinforce the notion that biallelic *BIRC3* inactivation should be considered as a high-risk CLL entity.

In summary, this work displays a comprehensive biological analysis of the impact of monoallelic and biallelic *BIRC3* lesions in del(11q) CLL patients by combining in vitro, ex vivo, and xenograft models. We show that monoallelic *BIRC3* deletion activates NF-κB signaling in del(11q) CLL cells, contributing to the pathobiology of this high-risk cytogenetic alteration. We also demonstrate that *BIRC3* mutation in the remaining allele of del(11q) CLL cells confers clonal advantage which could account for the negative predictive impact of *BIRC3* biallelic inactivation in CLL. Moreover, cells harboring these alterations could be therapeutically targeted with BCL2 inhibitors. Taken together, our results suggest that del(11q) CLL patients harboring *BIRC3* mutations should be considered as a CLL subgroup at a high risk of progression that might benefit from venetoclax-based therapies.

## Supplementary information

Supplementary Information

Reproducibility checklist
