# Supplementary material for: Biological significance of monoallelic and biallelic BIRC3 loss in del(11q) chronic lymphocytic leukemia progression
Source: Blood Cancer J. 2021 Jul 9;11(7):127. doi: 10.1038/s41408-021-00520-5 (PMC8270906; doi:10.1038/s41408-021-00520-5)
Supplement: Supplementary file 1 — Supplementary Information [file 41408_2021_520_MOESM1_ESM.docx]

**SUPPLEMENTARY INFORMATION**

**Biological significance of monoallelic and biallelic *BIRC3* loss in del(11q) chronic lymphocytic leukemia progression**

Miguel Quijada-Álamo, María Hernández-Sánchez, Ana-Eugenia Rodríguez-Vicente, Claudia Pérez-Carretero, Alberto Rodríguez-Sánchez, Marta Martín-Izquierdo, Verónica Alonso-Pérez, Ignacio García-Tuñón, José María Bastida, María Jesús Vidal-Manceñido, Josefina Galende, Carlos Aguilar, José Antonio Queizán, Isabel González-Gascón y Marín, José-Ángel Hernández-Rivas, Rocío Benito, José-Luis Ordóñez and Jesús-María Hernández-Rivas

**SUPPLEMENTARY METHODS**

**Cell lines, culture conditions, drugs and reagents**

The human CLL-derived cell lines HG3 and MEC1 were purchased from DMSZ (Deuthche Sammlung von Mikroorganismen and Zellkulturen). HG3 cells were cultured in RPMI 1640 medium (Life Technologies) supplemented with 15% Fetal Bovine Serum (FBS), 1% glutaMAX and 1% penicillin/streptomycin (Life Technologies). MEC1 cells were cultured in cultured in Iscove’sMDM medium (Lonza) supplemented with 10% FBS, 1% glutaMAX and 1% penicillin/streptomycin. HS-5 bone marrow stromal cells for *ex vivo* co-cultures were purchased from ATCC and HEK 293T cells for lentiviral production were obtained from DMSZ. Both cell lines were maintained in DMEM (Life Technologies) supplemented with 10% FBS, 1% glutaMAX and 1% penicillin/streptomycin. All cell lines were incubated at 37ºC in a 5% CO2 atmosphere. The presence of mycoplasma was tested frequently with MycoAlert kit (Lonza), only using mycoplasma-free cells in all the experiments carried out.

Venetoclax (ABT-199) and birinapant were obtained from LC Laboratories, A-1331852 (BCL-xL inhibitor),S63845 (MCL1 inhibitor) and SMI1 (NIK inhibitor) were purchased from Selleckchem and fludarabine was from Sigma. All drugs were resuspended in DMSO (Sigma).

**Next-generation sequencing**

A targeted-capture next-generation sequencing (NGS) strategy was used to analyze the mutational status of *BIRC3* in CLL patients, as well as other 57 genes previously reported as mutated in CLL or involved in the disease pathogenesis (1, 2) following the approach of Agilent SureDesign. Sequence data generated by Illumina NextSeq 500 was analyzed by an in-house bioinformatic pipeline as previously reported (3). Supplementary Table S2 shows the list of mutations of each CLL patient.

In addition, targeted-capture NGS data was also used to assess whether or not the deleted region on del(11q) detected by FISH included *BIRC3* gene in CLL patients. The mean coverage depth of each individual exon target was first normalized in a set of samples without deletion on 11q by FISH using the total read number of each sample. The mean coverage of all these samples was used as the reference. To detect del(11q) and assess which genes were involved, the normalized coverage of exons of genes located in 11q (*NXF1*, *BIRC3* and *ATM*) from each study sample was compared to the mean coverage of the same target in the reference file generated above. Copy number variations (CNVs) were called using fixed thresholds representing the log2 ratio of mean coverage of testing to that of reference. A log2 ratio <-0.5 suggested a heterozygous deletion of 11q. Therefore, these analyses allowed us to determine whether *BIRC3* was deleted or not in CLL samples with del(11q). HG3-del(11q) cell line, in which *ATM* and *BIRC3* is monoallelic deleted, was analyzed as a control positive since the deletion size was known (Supplementary Fig. S1). This method was based on a previously published analysis to detect deletions from targeted-capture NGS data (4) and has been also used to determine CNVs in inherited platelet disorders (5).

**Fluorescence *in situ* hybridization (FISH)**

Interphase FISH was performed in primary CLL PBMCs or CRISPR/Cas9-engineered cell lines using commercially available probes for the 13q14, CEP12, 11q22/ATM and 17p13/P53 (Vysis, Abbott Laboratories, IL, USA) as previously described (6). Signal screening was carried out in at least 200 and the cut-off for positive cases was set at >10% events.

**Subcellular fractionation and western blot**

Subcellular fractionation was performed using the Subcellular Protein Fractionation Kit (ThermoFisher Scientific) according to the manufacturer’s instructions. For whole cell-lysates, cells were washed with PBS and lysed in ice-cold lysis buffer (140 mmol/l NaCl, 50 mmol/l EDTA, 10% glycerol, 1% Nonidet P-40, 20 mmol/l TrisHCl pH 7) containing protease inhibitors (cOmplete^TM^, Roche) and phosphatase inhibitors (PhosSTOP^TM^, Roche). The following primary antibodies purchased from Cell Signaling Technologies were used in the western blot experiments: anti-BIRC3 (#3130, Rabbit), anti-NF-κB2 (#4882, Rabbit), anti-p- NF-κB2 (#4810, Rabbit) anti-NIK (#4994, Rabbit), anti-RelB (#10544, Rabbit), anti-p-IKKα/β (#2697, Rabbit), anti- NF-κB1 (#13586, Rabbit), anti-BCL2 (#2872, Rabbit), anti-BCL-xL (#2762, Rabbit), anti-MCL1 (#94296, Rabbit), anti-BIM (#2933, Rabbit), anti-BAK (#12105, Rabbit), anti-BAX (#5023, Rabbit) anti-NOXA (#14766, Rabbit), anti- β-actin (#4967, Rabbit), anti-H3 (#4499, Rabbit) and anti-GAPDH (#5174, Rabbit). Horseradish peroxidase-linked anti-rabbit antibody (#7074, Cell Signaling Technologies) was used as secondary antibody at 1:5,000 dilution. Antibody signal was detected using ECL^TM^ Western Blotting Detection Reagents (RPN2209, GE Healthcare). Protein expression level was calculated by testing the ratios of each protein in relation to the loading control (β-actin, GAPDH or H3) using ImageJ software.

**MTT viability and growth assays**

Cell viability and proliferation was assessed using 3-(4,5-dimethylthiazol-2-yl)-2,5-diphenyltetrazolium bromide (MTT) colorimetric assay (Sigma-Aldrich). Cells were counted and seeded at a density of 1 x 10^4^ cells/well (for 72h experiments) or 2 x 10^4^ (for 48h experiments) in 96-well plates and treated with or without different drug treatments. At the time of analysis, cells were incubated for 2h with a 1:10 MTT solution and subsequently added 1:2 SDS-HCl in agitation for 6 hours. Absorbance was read on an Infinite® F500 Tecan plate reader (Tecan) at 570nm.

For the determination of the growth exponential curves of CRISPR/Cas9-edited clones, cells were seeded at a concentration of 4 x 10^4^ cells/mL and cell counts were assessed every 24 hours by Trypan Blue exclusion.

**Apoptosis and cell cycle analysis**

Apoptosis in response to drug treatment was measured by flow cytometry with annexin V-Dy634 (Immunostep) according to the manufacturer’s instructions. In brief, 3 x 10^5^ cells were seeded in 24-well plates and treated 48 hours with the drug concentration of interest, then they were labeled with annexin V and propidium iodide (PI). In parallel, cell distribution in the cell cycle phase was also analyzed measuring the DNA content by PI labeling after cell permeabilization.

***In vitro* clonal competition assays**

GFP- or RFP-tagged CRISPR/Cas9-generated clones were mixed at a 1:1 ratio and seeded at a density of 5 x 10^5^ cells/mL in 75 cm^3^ flasks. *In vitro* clonal evolution was assessed every 3-4 days by quantification of GFP+ and RFP+ population using a FACS Aria flow cytometer (BD Biosciences). Data was analyzed using FlowJo software.

**Subcutaneous xenograft experiments**

20 four-to-five-week-old female NOD/SCID/IL2 receptor gamma chain null (NSG) mice were used in a subcutaneous xenograft model to compare tumor volumes between HG3-edited clones. Tumor xenografts were induced by subcutaneous injection of cell suspensions containing 5 × 10^6^ cells in 0.2 ml of cellular medium into the mouse flank. Two groups of mice (n=10) were injected in the right flank with either HG3 WT clones or HG3 *BIRC3* mutated clones. Tumor volumes were measured with a caliper every 2-3 days. Volumes were calculated using the formula: a2bπ/2, where a and b are the 2 maximum diameters. Mice were sacrificed by anesthesia overdose 18 days after cell injection, when tumors were weighted and collected for histopathological analysis.

**Immunohistochemistry**

Tumor processing and immunohistochemistry (IHC) were performed as previously described (7) using an anti-NF-κB2 antibody (#3017, Cell Signaling Technologies). An experienced pathologist from the Molecular Pathology Unit of the Cancer Research Center of Salamanca supervised the analysis of the samples under a Leica microscope for the evaluation of p52 expression in the subcutaneous and intravenous xenografts.

**Supplementary References**

1. Landau DA et al. Mutations driving CLL and their evolution in progression and relapse. Nature. 2015;526(7574):525–30.

2. Puente XS et al. Non-coding recurrent mutations in chronic lymphocytic leukaemia. Nature. 2015;526(7574):519–24.

3. Quijada-Álamo M et al. CRISPR/Cas9-generated models uncover therapeutic vulnerabilities of del(11q) CLL cells to dual BCR and PARP inhibition. Leukemia. 2020;34(6):1599–612.

4. Feng Y, Chen D, Wang G-L, Zhang VW, Wong L-JC. Improved molecular diagnosis by the detection of exonic deletions with target gene capture and deep sequencing. Genet Med. 2015;17(2):99–107.

5. Bastida JM et al. Introducing high-throughput sequencing into mainstream genetic diagnosis practice in inherited platelet disorders. Haematologica. 2018;103(1):148–62.

6. González MB et al. The value of fluorescence in situ hybridization for the detection of 11q in multiple myeloma. Haematologica. 2004;89(10):1213–8.

7. Ordóñez JL et al. The PARP inhibitor olaparib enhances the sensitivity of Ewing sarcoma to trabectedin. Oncotarget. 2015;6(22):18875–90.

**Supplementary Table S1. sgRNA sequences and PCR primers of sgRNA target sites.**

| **Target** | **Forward (5’-3’)** | **Reverse (5’-3’)** |
| --- | --- | --- |
| *BIRC3* exon 2 sgRNA1 | CACCG**ATTGAGCAATTGGGAACCGA** | AAAC**TCGGTTCCCAATTGCTCAAT**C |
| *BIRC3* exon 2 sgRNA2 | CACCG**GAGAGTTTGAATAAGAGCCA** | AAAC**TGGCTCTTATTCAAACTCTC**C |
| *BIRC3* exon 7 sgRNA3 | CACCG**ATTAATCCGGAAGAATAGAA** | AAAC**TTCTATTCTTCCGGATTAAT**C |
| Control sgRNA1 | CACCG**ACGGAGGCTAAGCGTCGCAA** | AAAC**TTGCGACGCTTAGCCTCCGT**C |
| *BIRC3* exon 2 (PCR) | ACGACTTGTCATGTGAACTGTACC | GCAGATTCAGTTTCTTACCCACATA |
| *BIRC3* exon 7 (PCR) | GAGACACCCCTAAACCTAGCA | GCCAAATACTCATTTCAAGGCAAC |

**Supplementary Table S2. Biological characteristics of CLL patients.**

| **Sample ID** | **Sex** | **IGHV status** | **Cytogenetics (FISH)** | **% del(11q)** | ***BIRC3* deleted** | ***BIRC3* mutations** | **Other known mutations** | **Sample used in** |
| --- | --- | --- | --- | --- | --- | --- | --- | --- |
| **ID-01** | M | UM | del(11q), del(13q), trisomy 12 | 65% | Yes |  | NOTCH1, ATM, ZNF292 | Fig. 3a, b |
| **ID-02** | F | M | del(11q), del(13q) | 27% | Yes |  | POT1 | Fig. 3a |
| **ID-03** | M | UM | del(11q) | 75% | Yes |  | ATM, ARID1B | Fig. 3a |
| **ID-04** | M | UM | del(11q), del(13q) | 95% | Yes |  | XPO1, FUBP1 | Fig. 3a, b |
| **ID-05** | M | UM | del(11q), del(13q) | 60% | Yes |  | SF3B1, ATM, DDX3X | Fig. 3a |
| **ID-06** | F | NA | Normal |  |  |  | ZNF292 | Fig. 3a |
| **ID-07** | M | NA | del(11q), del(13q) | 80% | No |  | SF3B1, ATM, ARID1A, ZC3H18 | Fig. 3a, b |
| **ID-08** | F | UM | del(13q) |  |  |  | SF3B1, NOTCH1, MED12, RPS15 | Fig. 3a |
| **ID-09** | F | UM | trisomy 12 |  |  |  | ATM, TRAF3 | Fig. 3a |
| **ID-10** | F | NA | del(11q), del(13q) | 90% | Yes |  | MAP2K1, BRAF, DDX3X | Fig. 3a, b |
| **ID-11** | M | M | del(11q) | 95% | No |  | SF3B1, EGR2 | Fig. 3a, b |
| **ID-12** | M | UM | del(13q) |  |  |  | NOTCH1, XPO1, MGA | Fig. 3a |
| **ID-13** | M | M | del(13q) |  |  |  | MYD88 | Fig. 3a |
| **ID-14** | M | M | trisomy 12 |  |  | p.E433fs (8%); p.Q547fs (6%) | FBXW7, IGLL5, ARID1B | Fig. 3a |
| **ID-15** | F | M | Normal |  |  |  |  | Fig. 3a |
| **ID-16** | M | M | del(13q) |  |  |  |  | Fig. 3a |
| **ID-17** | M | UM | Normal |  |  |  | SF3B1, ATM, POT1 | Fig. 3a |
| **ID-18** | M | UM | del(11q), del(13q) | 80% | Yes |  | ATM | Fig. 3a |
| **ID-19** | M | UM | del(11q), del(13q), trisomy 12 | 70% | Yes | p.C581G (62%) | XPO1, NOTCH1, CHD2, SETD2, ZMYM3, ZNF292 | Fig. 3a, b |
| **ID-20** | F | M | trisomy 12 |  |  | p.C557fs (24%) | FBXW7, KRAS, KLHL6 | Fig. 3a |
| **ID-21** | M | UM | del(11q), del(13q) | 97% | No |  | TRAF3 | Fig. 3a, b |
| **ID-22** | M | NA | del(11q), del(13q) | 85% | Yes |  | SF3B1, ATM, CHD2, ASXL1, ENTPD4 | Fig. 3a |

**Supplementary Table S3. List of mutations detected in primary CLL cases used in *ex vivo* experiments.**

**Supplementary Figure S1**

**Detection of 11q deletion in cell lines and primary CLL samples using targeted-capture NGS data.** Profile of log2 ratios of normalized mean coverage of individual exon target of genes located in 11q (*NFX1*, *BIRC3* and *ATM*) to that of the reference, was plotted against the target. The x-axis shows the targets in the panel plotted by relative genome order. The y-axis corresponds to the log2 ratio of the mean coverage of testing to that of reference. A log2 normalized coverage ratio <-0.05 indicates a heterozygous deletion of chr11 (11q region). This analysis allowed us to determine whether *BIRC3* is deleted or not in CLL samples with del(11q). Left panel shows graphs from HG3 parental cell line (therefore, without del(11q)), HG3-del(11q) generated by CRISPR/Cas9, confirming the presence of del(11q) encompassing *ATM* and *BIRC3,* and MEC1 parental cell line harboring a monoallelic deletion of *BIRC3* (exons 7-9). Right panel shows three representative graphs from primary CLL samples: ID-06 without del(11q); ID-10 with del(11q) including loss of *ATM* and *BIRC3*; ID-11 with del(11q) including only loss of *ATM* (*BIRC3* undeleted). The presence of del(11q) was validated by FISH in cell lines as well as primary CLL samples.

**Supplementary Figure S2**

**Generation of isogenic MEC1 CLL derived cell lines harboring *BIRC3* mutations using the CRISPR/Cas9 system.** Upper panel: scheme of CRISPR/Cas9 induction of *BIRC3* mutations in MEC1 cells. sgRNA targeting *BIRC3* was transduced in MEC1 parental cells (which harbor a *BIRC3* monoallelic deletion; *BIRC3*^DEL/WT^), generating MEC1-edited clones with *BIRC3* biallelic inactivation (*BIRC3*^DEL/MUT^). Lower panel: western blot analysis of MEC1-edited cell lines harboring *BIRC3* loss-of-function mutations. β-actin was used as loading control.

**Supplementary Figure S3**

**NF-κB-related effects of *BIRC3* deletion and/or mutation in CRISPR/Cas9-edited HG3 and MEC1 CLL cell lines. (A)** ELISA measurement of relative NF-κB2 p52 and RelB DNA-binding activity in nuclear extracts from HG3^WT^ and HG3 *BIRC3*^MUT^ clones. Bars represent the mean ± SD. **(B)** Quantification of proteins analyzed by western blot in Fig. 2b relative to loading control. Data is summarized as the mean ± SD of three independent clones. **(C)** ELISA measurement of relative NF-κB family transcription factor DNA-binding activity in nuclear extracts from MEC1-edited clones. Left panel shows DNA-binding activity of NF-κB transcription factors involved in the canonical signaling (p65, c-Rel and p50). Right panel displays the DNA-binding activity of non-canonical NF-κB transcription factors (p52 and RelB). Data are represented as the mean ± SD. **(D)** Whole-cell, cytoplasmic and nuclear lysates of CRISPR/Cas9-edited MEC1 clones analyzed by immunoblotting for NIK and NF-κB2 (p100/p52) protein. GAPDH was used as loading control.

**Supplementary Figure S4**

**Impact of BIRC3-mediated non-canonical NF-κB signaling activation in BCL2-family members. (A)** Quantification of proteins analyzed by western blot in Fig. 2c relative to loading control. Data is summarized as the mean ± SD of three independent clones. **(B)** Western blot analysis of HG3^WT^, HG3-del(11q) and HG3-del(11q) *BIRC3*^MUT^ cells treated with the indicated doses of NIK SMI1. Whole-cell lysates were extracted 24 hours after treatment and probed for NF-κB2 (p100/p52), BCL2 and BCL-xL proteins. β-actin was used as loading control.

**Supplementary Figure S5**

**Response to ibrutinib and fludarabine of HG3 CRISPR/Cas9-generated clones. (A)** Dose-response curves of HG3^WT^, HG3-del(11q) and HG3-del(11q) *BIRC3*^MUT^ clones treated with ibrutinib. Cell viability was assessed by MTT assay after 72 hours and surviving fraction is expressed relative to DMSO control. Data is summarized as mean ± SEM. **(B)** Cell cycle analysis of CRISPR/Cas9-edited clones after 48 hours 5 μM fludarabine exposure. SubG_0_ peak is indicative of the presence of apoptotic cells. **(C)** Representative plots of annexin V/PI stained HG3^WT^, HG3 *BIRC3*^MUT^, HG3-del(11q) *BIRC3*^MUT^ and HG3 *TP53*^MUT^ cell lines 48 hours after 5 μM fludarabine exposure. Fludarabine induced apoptosis is shown by the presence of annexin V+ cells.

**Supplementary Figure S6**

**Effects of BIRC3 deletion, mutation or inhibition in growth, viability and cell cycle of HG3 and MEC1 CLL cell lines. (A)** Growth rate assessment of HG3^WT^, HG3-del(11q) and HG3-del(11q) *BIRC3*^MUT^ cells (left panel), or HG3^WT^ and HG3 *BIRC3*^MUT^ cells (right panel), by trypan blue exclusion. Data were fitted in an exponential growth equation, and time point values are presented as the mean ± SEM. **(B)** Cell cycle phase distribution of HG3-edited cell clones. Data represent the mean values ± SD of three independent experiments. **(C)** HG3^WT^ cells were treated with the BIRC2/BIRC3 inhibitor birinapant (2.5 μM) and viability was assessed at 6 days by MTT**.** Absorbance values are represented normalized with the control (DMSO) condition. Data are summarized as mean ± SD. **(D)** Effect of *BIRC3* mutation in the proliferation of MEC1 cells after 72 hours. MTT absorbance values are represented normalized with the MEC1^DEL/WT^ clones. Data are represented as the mean ± SD.

**Supplementary Figure S7**

**Implications of *BIRC3* mutations in tumor growth of *in vivo* subcutaneous xenografts. (A)** Tumor growth evolution (mm^3^) following subcutaneous cell injection of HG3^WT^ and HG3 *BIRC3*^MUT^ cells (*n* = 10 mice/group). The plot displays mean ± SD values over 17 days. **(B)** Immunohistochemical analysis of NF-κB2 (p52) expression of tumor xenografts.

**Supplementary Figure S8**

**Impact of *BIRC3* deletion and/or mutation in the non-canonical NF-κB signaling.** Left panel: Functional BIRC3^WT^ protein targets NIK for proteasomal degradation in the cytoplasm of CLL cells, keeping the non-canonical NF-κB signaling inactive. Right panel: when *BIRC3* is deleted through del(11q) or truncated through loss-of-function mutations, NIK is stabilized in the cytoplasm activating downstream signaling and ultimately leading to RelB-p52 nuclear translocation and overexpression of anti-apoptotic BCL2 family members.
